# Occurrence of Terrifying Nightmares after Few Days of Mirtazapine Use in Elderly Patients

**DOI:** 10.1155/2023/8843206

**Published:** 2023-04-04

**Authors:** Liliana Dell'Osso, Primo Lorenzi, Benedetta Nardi, Barbara Carpita, Francesca Benedetti, Ivan Mirko Cremone

**Affiliations:** Department of Clinical and Experimental Medicine, University of Pisa, Pisa, Italy

## Abstract

*Introduction*. Sleep disturbance and insomnia are some of the most frequent complaints in patients suffering from depression. Some common antidepressant with excitatory effects may worsen sleep qualities, whereas others (like mirtazapine), thanks to their antihistaminergic action, are associated with sedative properties and can quickly improve sleep quality. In the case of mirtazapine, even if its mechanisms of action on sleep remain controversial, beneficial changes in sleep pattern may be observable since the first dose and are associated with a faster onset of the antidepressive action. *Case Presentation*. Despite these documented beneficial effects, we reported five cases of elderly patients (age ranging from 69 to 79) with various diagnoses and comorbidities (severe or recurrent depression, general anxiety disorder, borderline personality disorder, and Parkinson's disease) assessed during clinical daily routine for whom the use of mirtazapine was linked to the onset of nightmares so impressive and dramatic that made it necessary to interrupt the treatment. *Discussion*. This peculiar side effect is still scarcely documented, and the literature on this topic remains conflicting; however, considering that the cases were collected in a short range of time, the exacerbation of nightmares caused by mirtazapine may be more frequent than previously believed. Furthermore, some common features shared by all the cases reported have been highlighted such as the onset of the nightmares being chronologically associated with the initiation of the therapy with mirtazapine, the disappearance with the interruption, the similar age range of all, and the occurrence of the episodes described during fall season.

## 1. Introduction

Depression is a severe and common mental disorder worldwide, with an estimated prevalence of 3.8% in the general population and 5.0% among adults [1]. Disrupted sleep and insomnia are frequent complaints in patients suffering from depression, and, on the other end, they are also risk factors for the development of a depressive episode [2]; unfortunately, sleep disturbances are often part of the residual symptoms linked to incomplete remission [3]. It is common knowledge that some antidepressants, specifically those with excitatory effects (such as fluoxetine, venlafaxine, and bupropion), may worsen sleep quality through the activation of serotonergic 5-HT_2_ receptors and increased noradrenergic and dopaminergic neurotransmission [3]. On the other hand, antidepressants with an antihistaminergic action (such as mirtazapine) or a strong antagonistic function on 5-HT_2_ receptors (like trazodone) are associated with sedative properties and can quickly improve sleep quality or even cause sedation and hypersomnia. In some cases, the change in sleep pattern may be observable since the first dose [4], particularly with the use of mirtazapine, and it has been associated with a quicker onset of the antidepressant action [5].

Mirtazapine is a tetracyclic antidepressant belonging to the piperazine group, with a known dual mode of action; on one hand, it increases noradrenergic and serotonergic transmission, antagonizing the adrenergic a-autoreceptors and a_2_-heteroreceptors (acting as a noradrenergic and specific serotonergic antidepressant (NaSSA)); on the other hand, it is known to enhance the release of serotonin via the stimulation of 5HT_1_ receptor and the block of 5HT_2_ and 5HT_3_ receptors [6]. This dual enhancement of noradrenergic and 5HT_1_ receptor activity is thought to underlie its antidepressant properties and may be responsible for its rapid onset of action. Mirtazapine is extensively metabolized in the liver by the cytochrome (CYP) P450 isoenzymes C1A2, CYP2D6, and CYP3A4, reporting limited drug-to-drug interactions, and thanks to its pharmacodynamical properties, a steady-state concentration is usually reached after 4 days of a daily dose [7]. Mirtazapine could be a useful drug choice for the treatment of major depression, fulfilling the needs for tolerability (dry mouth, sedation, and increased appetite are the most common adverse effects whereas it seems to have no side effects on sexual drive) and safety in overdose [7]. In particular, its broad clinical use as an antidepressant is also linked to its anxiolytic and sleep-improving properties for the management of depression with insomnia [8, 9]. Despite these undoubtedly beneficial effects, we reported five cases of elderly patients assessed during our clinical daily routine, among whom the use of mirtazapine was linked to the onset of nightmares. Noticeably, the reported nightmares were so impressive and dramatic that made it necessary to interrupt the treatment. A search in the recent scientific literature showed a very limited number of previous reports about this peculiar side effect; for example, the study of Sudha et al., Mathews et al., Dang et al., and Menon and Madhavapuri [7, 10–12] describes the case of a 48-year-old male with difficulty in the initiation and maintenance of sleep, fluctuating anxiety, and global functioning impairment in all work-related activities. The patient was prescribed mirtazapine 7.5 mg at night for two days and 15 mg from the third day onwards. Within three days, he reported total insomnia and sudden awakening with profuse sweating in the middle of the night, all associated with terrific dreams. After he was advised to stop mirtazapine immediately, the patient reported having no nightmares from the fourth day on, with altogether good sleep. Two more cases were from India: one regarding a 21-year-old man with depressive symptoms treated with 15 mg of mirtazapine and the other describing a female of the same age diagnosed with major depression and taking half dose of the same therapy; the last one was from Philadelphia and was about a 52-year-old man with depressive symptoms, treated with 15 mg of mirtazapine (see Table 1) [7, 10–12].

Thus, the aim of this work is to describe five cases of depression with treatment-emergent nightmares induced by mirtazapine that came to our attention and to discuss its possible underpinnings.

## 2. Case Presentation

For a summary of the cases presented, see Table 2.

### 2.1. Case 1

J.K., a 71-year-old woman with a long history of severe depression, comes to our clinical attention with a previous treatment based on antidepressants (venlafaxine and fluoxetine), pregabalin, and benzodiazepines. In order to address her severe and persisting insomnia problems, we decided to introduce mirtazapine 30 mg/daily. In the very next few days, she reported a significant improvement of the sleeping time but also the sudden and progressive appearance of nightmares, so disturbing that the patient autonomously decided to stop the treatment. Soon after the discontinuation of the drug, the nightmares disappeared and the insomniac pattern came back.

### 2.2. Case 2

X.Y., a 69-year-old woman with a diagnosis of borderline personality disorder in comorbidity with depressive and dissociative episodes, was in treatment with SSRI and benzodiazepines. Mirtazapine 45 mg once a day was introduced with the aim to reach a greater control over anxious symptoms and disrupted sleep. While the reduction of the anxiety was significant, at the same time, the patient started to experience dramatic nightmares. Considering the good clinical results on the depressive and anxious symptomatology, X.Y. tried to endure the nightmares and continued the treatment. However, over a relatively short lapse of time, the nightmares and related parasomnias grew so much in intensity that a discontinuation of mirtazapine became necessary. After removing the use of mirtazapine, the nightmares quickly disappeared.

### 2.3. Case 3

H.I., a 79-year-old man without a history of psychiatric disorders during lifetime, was in treatment with dopaminergic drugs for a Parkinson's disease during the last 5 years. He came to our attention after the appearance of a depressive mood, jealous ideation, and insomnia. The introduction of mirtazapine improved the mood and resolved the insomnia, but at the same time, nightmares appeared, gaining progressive strength and relevance. As in the previous cases, after some weeks, this side effect became so unbearable to require the suspension of mirtazapine. A few days after, the nightmares disappeared.

### 2.4. Case 4

L.Z., a 75-year-old woman, was in treatment with paroxetine 20 mg daily and mirtazapine 15 mg daily for a recurrent depression in a borderline personality disorder. After increasing the dosage of mirtazapine to 30 mg, the patient experienced a huge number of terrific nightmares. The patient managed to continue the therapy only for 5 days before autonomously deciding to interrupt all the prescribed medications with angry feelings towards the therapy. In this case, the nightmares disappeared since the first night after the discontinuation.

### 2.5. Case 5

B.S., a 72-year-old man with a general anxiety disorder with recurrent depressive episodes since his early adulthood, was in treatment with SSRI and low doses of benzodiazepines. He came to our attention for the onset of a new major depressive episode associated with anxious symptoms and a critical disruption of the sleep pattern, which led to the use of mirtazapine 30 mg daily. Although he reported a notable improvement of depressive symptoms, anxiety, and sleep pattern, after a short period of time, he started to experience terrifying dreams, becoming more and more frequent and devastating. Such nightmares led to a progressive increase of anxiety symptoms, and at least one time, a full-blown panic attack happened during the night. Even in this case, mirtazapine's discontinuation became necessary and led to the disappearance of any sleep disturbance and of nighttime panic attacks.

## 3. Discussion

On the basis of the current literature, mirtazapine's mechanisms of action on sleep remain controversial. While its ability to block *α*_2_-autoreceptors could lead to impaired REM sleep and to a disruption of sleep continuity, its inhibition on the 5-HT_2_ and, to a lesser extent, H_1_ receptors may tend to promote sleep [13, 14]. All the cases reported herein share many common features. Firstly, in each one, the onset of the nightmares was chronologically associated with the initiation of the pharmacological therapy with mirtazapine (side effect appeared in a space encompassed between 48 hours and a week), while their disappearance shortly followed mirtazapine interruption (within 48 hours after the discontinuation). Secondly, all the patients were elderly subjects of a similar age range; lastly, all those episodes occurred during fall season. It is to notice that in none of the cases, there was a wash-out period but only a switch or an add-on passage. Considering that the cases were collected in a short range of time, it could be hypothesized that the exacerbation of nightmares caused by mirtazapine may be more frequent than previously believed. Unfortunately, to date, the literature on this topic remains scarce and conflicting. Some previous studies, comparing mirtazapine with fluoxetine and paroxetine, other common antidepressants, reported instead less nocturnal disturbance and better sleep efficiency with the use of mirtazapine [15–17]. Nightmares can be considered a “REM sleep-related behavior disturbance” (RBD) and thus a kind of parasomnia that may include the loss of the normal muscle hypotonia present during REM sleep, portrayed by a dream enactment and the appearance of terrific and realistic issues [17]. Therefore, hypothesizing that nightmares can be caused generically by an alteration or even repression of REM sleep, a great contradiction arises, since mirtazapine, unlike many other antidepressants, commonly has no repressive effect on REM sleep [18].

Literature data suggest that nightmares can also be observed in the general population, often reaching clinical relevance in two conditions: during Parkinson's disease or parkinsonism [19, 20] and in posttraumatic stress disorder (PTSD) [21, 22]. In the third case of our series, the presence of mirtazapine-induced nightmares in a patient with Parkinson's disease could be connected with alterations of the cholinergic structures, in the sense of a reduced function. The pathophysiological underpinnings may be linked to a disruption of the cholinergic nucleus subcoeruleus [23], eventually attributable to the pressure exerted by a noradrenergic or dopaminergic hyperactivity related to the use of dopaminergic drugs (related to Parkinson's disease) and/or to the noradrenergic effects on mirtazapine. In this framework, further studies, when evaluating the mechanisms of action of mirtazapine on sleep, should focus on clarifying the role of an altered function of nucleus subcoeruleus, which could be related to Parkinson's disease, prolonged treatment with anticholinergic drugs, and advanced age [22, 24]. Moreover, abnormal aminergic pressure on these structures, which often occurs during treatments with aminergic drugs, in chronic stress and chronic alarm conditions associated mood disorders, borderline personality disorder, and PTSD, could further impair the function of nucleus subcoeruleus [25]. These mechanisms may represent two sides of the same neuropathological pathway, which ultimately would lead to an increased presentation of nightmares with the use of mirtazapine. Globally, further studies in wide samples and with a longitudinal design are needed to clarify the positive and negative impacts of mirtazapine on nightmares in different populations.

## 4. Limits

This work should be considered in light of some limitation. Firstly, none of the patient was in therapy exclusively with mirtazapine, but they were following a polytherapy. Secondly, none of the patient underwent an EEG (electroencephalogram) around the period of appearance/disappearance of nightmares. Lastly, the nightmares were evaluated exclusively with clinical interview, without the use of any scale.

## 5. Conclusions

In this paper, we reported five cases of treatment-emergent nightmares induced by mirtazapine in elderly patients with various diagnosis and comorbidities. To this date, the available literature on mirtazapine's mechanisms of action on sleep remains controversial, and the researches on this peculiar side effect are still in their infancy. Even if the data collected rightfully lead to the hypothesis that the exacerbation of nightmares caused by mirtazapine may be more frequent than previously believed, undoubtedly, further studies are needed on this topic.

## Figures and Tables

**Table 1:** Summary table of previous cases reporting the same side effect.

Citation	Clinical condition	Sex/age	Drug/dose	Inference
Sudha et al. [7]	Anxious symptoms and insomnia	M/48	Mirtazapine 15 mg	Nightmares
De Boer [8]	Depressive symptoms	M/52	Mirtazapine 15 mg	Nightmares
Cipriani et al. [9]	Depressive symptoms	M/21	Mirtazapine 15 mg	Nightmares
Mathews et al. [10]	Major depression	F/21	Mirtazapine 7.5 mg	Nightmares

**Table 2:** Summary table of the cases presented.

Citation	Clinical condition	Sex/age	Drug/dose	Inference
Ibidem	Severe depression	F/71	Mirtazapine 30 mg	Nightmares
Ibidem	Depressive and dissociative episodes in a BPD	F/69	Mirtazapine 45 mg	Nightmares
Ibidem	Depressive mood, jealous ideation, and insomnia in patient with Parkinson's disease	M/79	Mirtazapine 30 mg	Nightmares
Ibidem	Recurrent depression in a BPD	F/75	Mirtazapine 30 mg	Nightmares
Ibidem	General anxiety disorder with general anxiety disorder recurrent depressive episodes	M/75	Mirtazapine 30 mg	Nightmares

## Data Availability

All data generated or analyzed during this study are included in this published article.
